# Eating disorder behaviours amongst adolescents: investigating classification, persistence and prospective associations with adverse outcomes using latent class models

**DOI:** 10.1007/s00787-016-0877-7

**Published:** 2016-06-24

**Authors:** Nadia Micali, N. J. Horton, R. D. Crosby, S. A. Swanson, K. R. Sonneville, F. Solmi, J. P. Calzo, K. T. Eddy, A. E. Field

**Affiliations:** 1 Department of Psychiatry, Icahn School of Medicine at Mount Sinai, New York, NY USA; 2 Mindich Child Health and Development Institute, Icahn School of Medicine at Mount Sinai, New York, NY USA; 3 Institute of Child Health, University College London, London, UK; 4 Department of Mathematics and Statistics, Amherst College, Amherst, MA USA; 5 Department of Biomedical Statistics, Neuropsychiatric Research Institute, Fargo, ND USA; 6 Department of Psychiatry and Behavioral Science, University of North Dakota School of Medicine and Health Sciences, Fargo, ND USA; 7 Department of Epidemiology, Harvard T. H. ChanSchool of Public Health, Boston, USA; 8 Department of Epidemiology, Erasmus MC, Rotterdam, Netherlands; 9 Human Nutrition Program, Department of Environmental Health Sciences, University of Michigan School of Public Health, Ann Arbor, MI USA; 10 Division of Psychiatry, University College London, London, UK; 11 Division of Adolescent Medicine, Department of Medicine, Boston Children’s Hospital and Harvard Medical School, Boston, MA USA; 12 Department of Psychiatry, Harvard Medical School, Boston, MA USA; 13 Eating Disorders Clinical and Research Program, Massachusetts General Hospital, Boston, MA USA; 14 Department of Epidemiology, Brown University School of Public Heath, Providence, RI USA

**Keywords:** ALSPAC, Adolescent, Eating disorders, Latent class

## Abstract

Diagnostic criteria for eating disorders (ED) remain largely based on clinical presentations, but do not capture the full range of behaviours in the population. We aimed to derive an empirically based ED behaviour classification using behavioural and body mass index (BMI) indicators at three time-points in adolescence, and to validate classes investigating prospective associations with adverse outcomes. Adolescents from the Avon Longitudinal Study of Parents and Children (ALSPAC) provided data on ED at age 14 (*n* = 6615), 16 (*n* = 5888), and 18 years (*n* = 5100), and had weight and height measured. Psychological and behavioural outcomes were assessed at 15.5/16 and 17.5/18 years. We fit gender- and age-stratified latent class models, and employed logistic regression to investigate associations between classes and later outcomes. One asymptomatic and two symptomatic (largely representing higher and lower frequency ED behaviours) classes were observed at each time-point, although their relative prevalence varied by age and gender. The majority of girls in symptomatic classes remained symptomatic at subsequent assessments. Girls in symptomatic classes had higher odds of subsequent anxiety and depressive disorders, binge drinking, drug use, and deliberate self-harm. Data analyses were underpowered amongst boys. The presence of two symptomatic classes (characterised by different ED behaviour frequency) and their prospective association with adverse outcomes suggest a need to refine diagnostic thresholds based on empirical data. Despite some instability of classes, particularly in mid-adolescence, evidence that half of girls in symptomatic classes remained symptomatic suggests persistence of ED behaviours in adolescence, and highlights a need for early identification to reduce chronicity.

## Introduction

Eating disorders (ED) are common psychiatric disorders, which typically onset in adolescence [1], and are associated with high morbidity and/or mortality [2–4]. The Diagnostic and Statistical Manual for mental disorders 5th edition (DSM-5) [5] refined ED diagnostic categories, based on a consensus in the field that previous classifications did not fully capture diagnostic presentations as seen across clinical and community samples. DSM-5 has broadened ED diagnostic criteria, aiming to reduce the number of individuals with an ED who do not fit full-threshold diagnostic categories. Similar revisions are likely to be implemented in the 11th edition of the International Classification of Diseases (ICD) [6].

Despite these changes, ED diagnostic classification remains largely based on clinical presentations rather than empirical evidence. This is problematic for several reasons, first and foremost due to the potential lack of validity of such a classification across clinical and research practice [7]. Feinstein [8] noted that in medicine classification serves three main functions: denomination, i.e., assigning a common name to a combination of symptoms; qualification, i.e., adding descriptive features (for example characteristic symptoms, age of onset, etc.) to a category to enrich its utility; and prediction, i.e., being able to identify the expected course and outcome and likely response to treatment of a disorder. The inability of the DSM classification to capture the full range of ED and disordered eating (DE) in the community, and high documented rates of diagnostic crossover amongst ED have challenged the concurrent and predictive validity of available ED nosology [9, 10].

Leading up to the publication of DSM-5 several studies applied empirical methods to categorise ED. The majority of these have focused on adults, primarily from clinical samples. Whilst employing empirical classification methods in clinical samples can be useful, it nevertheless focuses on a selected subset of subjects, i.e., those who access treatment, and who might have higher psychiatric and physical comorbidity. Such an approach ignores a large group of individuals with ED and ED symptoms who do not access treatment [11, 12], limiting both the usefulness of the classification and potentially introducing bias in studying potential risk factors and outcomes. This limitation is particularly relevant to adolescence, given the time-lag between onset of symptoms (peaking in adolescence) and access to treatment [13], and the known lower treatment-seeking levels amongst adolescents and young adults [14, 15]. Thus, studying ED behaviour patterns in population-based samples is a useful approach to advancing our understanding of how these manifestations present in the community.

Adolescence is also a time of fluctuating levels of ED behaviours. A Norwegian study investigated empirically derived categories of ED behaviours in 623 adolescent girls and found that ED behaviour clusters were not stable over 7 years [16]. A larger study on young adults identified more stable DE patterns in older US female college students [17].

This suggests that adolescence might be a particularly important time to capture chronicity and natural fluctuations in DE, necessary to implement early intervention and prevention.

In a large sample of females in the United States, we previously identified four ED classes and found that those involving purging were at increased risk for binge drinking and drug use [18]. Using data obtained when adolescents were aged 13, when ED were rare, previously we empirically identified 3 DE patterns (binge eating/overeating, weight and shape concern and weight control behaviours and food restriction) among boys and girls from the Avon Longitudinal Study of Parents and Children-ALSPAC (the sample under study) [19]. Bingeing/overeating was strongly associated with higher functional impairment, family burden, and comorbid psychopathology. Bingeing/overeating and weight/shape concern and weight-control behaviours predicted higher BMI 2 years later, whereas food restriction predicted lower BMI 2 years later [19]. In the current study we aimed to extend our investigations using latent class methods by deriving an empirically based classification of DE across three time-points in adolescence (ages 14, 16 and 18) amongst boys and girls in a UK population-based cohort. We also aimed to determine transitions from each class to other classes across ages. Lastly, we determined the predictive validity of our empirical classification by investigating prospective associations between classes identified at 14 and 16 years of age and psychopathology (depression, anxiety), and problem behaviours (binge drinking, drug use and deliberate self-harm) approximately 2 years later.

## Methods

### Participants

The Avon Longitudinal Study of Parents and Children (ALSPAC) is a longitudinal, population-based, prospective study of women and their children [20, 21]. All pregnant women in the geographical area of Avon, UK, who were expected to deliver between 1st April 1991 and 31st December 1992 were invited to take part in the study. All women gave informed and written consent. Children (*n* = 14,676) from 14,451 pregnancies were enrolled; at 1 year 13,988 children were alive. At age 7 years (phase 2 and 3) 713 additional children were enrolled in the cohort [20]. Amongst twin-pairs, one twin per pair was randomly excluded for the present analyses due to non-independence.

We included adolescents based on participation to three waves of data collection: at child age 14, 16, and 18 years. At age 14, 16 and 18 years, respectively, 10,581, 9,702 and 9,505 adolescents were eligible for follow-up (i.e., had not withdrawn consent and were contactable for data collection when questionnaires were sent out) [20] and were sent questionnaires. Amongst these, 6,140 (58 %) 5,069 (52 %) and 3,228 (34 %), respectively, completed questionnaires on ED behaviours.

The study website contains details of all the data that is available through a fully searchable data dictionary: http://www.bris.ac.uk/alspac/researchers/data-access/data-dictionary.

### Disordered eating

Data on ED behaviours at each time-point were collected using questions from the Growing Up Today Study, adapted from the Youth Risk Behaviour Surveillance System questionnaire [22] and enquire about the previous year [23].Questions have been validated in a sample of adolescents in the Growing Up Today Study [24].

Purging was assessed by asking how often in the past year the adolescent made him/herself sick or used laxatives or other medicines to lose weight or avoid gaining weight.

Binge eating was assessed using a two-part question. Participants were first asked about the frequency during the past year of eating a very large amount of food; those who answered yes were directed to a follow-up question that asked whether they felt out of control during these episodes, like they could not stop eating even if they wanted. Binge eating was coded if adolescents answered yes to both questions.

Fasting was assessed by asking how often in the past year the adolescent had fasted (not eaten for at least a day) to lose weight or avoid gaining weight.

### Weight status

Weight and height were measured in clinic visits, at approximately age 14 (*n* = 6615), 16 (*n* = 5888), and 17.9 years (*n* = 5100). Age and gender adjusted BMI Z-scores according (using UK references) were derived from the Stata user-defined program “Z-anthro” [25, 26]. Normal weight/underweight, overweight, and obese categories were generated using age and gender specific cut-offs, derived for the UK according to International Obesity Task Force criteria [26]. Underweight was rare in the sample, therefore we did not separate this category from normal weight.

### Outcomes

Data on drug and alcohol use and depression were obtained from questionnaires at ages 16 and 18 years. Anxiety disorders and deliberate self-harm were assessed with validated semi-structured computerised interviews at face-to-face assessments at ages 15.5 and 17.5 years. At age 15.5 years the Development and Well-Being Assessment (DAWBA) interview [27] was used. At age 17.5 years the Clinical Interview Schedule-Revised (CIS-R) [28] was used to assess these outcomes.

Depression was measured using the short Moods and Feelings Questionnaire (sMFQ), a 13-item tool validated for adolescents [29, 30]. A binary variable indicating clinically relevant symptoms was derived using a cut-off of 8 [31], as previously described [12].

#### Drug use

Participants were asked about having used cocaine, crack, sedatives, opioids, inhalers, amphetamines, hallucinogens or other drugs in the previous year. Those reporting any drug use in the past year were classified as having used drugs.

#### Binge drinking

Adolescents’ drinking habits were assessed using the Alcohol Use Disorders Identification Test (AUDIT) [32] a short questionnaire to screen for problematic drinking. Binge drinking was defined as drinking ≥6 units of alcohol on one occasion at least monthly during the previous year.

#### Anxiety disorders

Diagnoses of anxiety disorders were obtained from the DAWBA [27, 33] anxiety disorders section at 15.5 years, and from the CIS-R [28] at 17.5 years. Participants were classified as having any anxiety disorders vs. none.

#### Deliberate self-harm (DSH)

Defined as having self-harmed at least once in the prior month (from the DAWBA) [27] at 15.5 years, and having reported any acts of self-harm during the previous year at 17.5 years.

### Statistical analyses

#### Latent class analysis

We undertook latent class analysis using a similar approach to that of Swanson et al. [18].

Latent class methods are used to classify response patterns using symptom indicators. Subjects are then assigned to a particular category (class) that their observed response patterns to symptom indicators indicate they are most likely to belong to. This method allows for partially observed participants to contribute to the model under a missing at random assumption [34].

The latent class models were fit using Mplus version 7.1. The indicators included in the model were BMI (underweight/normal weight, overweight, obese), purging (purging at least once a week, purging at least once a month but less than once/week, no purging), binge eating (overeating, binge eating, no binge eating/overeating), and fasting (fasting at least once a week, fasting at least once a month but less than once/week, no fasting) separately for each of the age levels. Consistent with previous recommendations [35], particular emphasis was given to the Consistent Akaike Information Criterion, minimum class sizes, and subject matter considerations to determine the optimal number of classes. Because DE is much less common in males than females, our analyses of males were very under-powered and should be considered as exploratory.

#### Transitions

To qualitatively assess changes in class membership over the three time points, gender-stratified pairwise cross-classifications were generated.

#### Predictive models

To assess the ability of the classifications to predict future dichotomous outcomes of interest (including binge drinking, anxiety disorders, depression, deliberate self-harm, and drug use), we fit a series of logistic regression models, which predicted the odds of the outcome as a function of latent class and baseline value of the outcome. All models were adjusted for age at assessment. The results of these models are described by an overall test for the latent class as well as pairwise comparison odds ratios and 95 % confidence intervals (comparing classes 2 and 3 (symptomatic classes) to class 1 (asymptomatic class), respectively).

#### Attrition

Prior reports [20] have described the missingness patterns of the ALSPAC study across adolescence, in particular non-response in ALSPAC is predicted by parental socio-economic status (SES) and child gender [20]. Availability of outcome data varied by assessment time-point [4] (for a detailed description). Partially observed data from subjects was accounted for by a missing at random (MAR) assumption [34]. Attrition at the later time-points was slightly higher in symptomatic classes; between 19.7 and 30.4 % of girls in symptomatic classes at age 14 or 16 years were missing at the following time-point, compared to 14.6–19.8 % of girls in the asymptomatic class.

#### Ethical approval

Ethical approval for the study was obtained from the ALSPAC Ethics and Law Committee and the Local Research Ethics Committees, all procedures were performed in accordance with the ethical standards laid down in the 1964 Declaration of Helsinki and its later amendments.

## Results

### Latent classes

Across genders and ages a three-class solution best fit our data (see Table S1). However, the three classes differed in terms of prevalence and probabilities of DE behaviours across gender and ages (see Table 1).

**Table 1:** Latent Class Models at three waves of data collection: proportions of girls endorsing each indicator across classes

Indicators	Disordered eating classes								
	Age 14 (*n* = 3,666)			Age 16 (*n* = 3,350)			Age 18 (*n* = 3,025)		
	Non- disordered (*n* = 3452, 94.1 %)	Overweight and overeating (*n* = 191, 5.1 %)	Weekly WCB and binge eating (*n* = 23, 0.6 %)	Non-disordered (*n* = 2915, 87.0 %)	Monthly WCB (*n* = 193, 5.8 %)	Weekly WCB (*n* = 242, 7.2 %)	Non-disordered (*n* = 2655, 87.8 %)	Monthly WCB (*n* = 275, 9.1 %)	Weekly WCB (*n* = 95, 3.1 %)
Weight									
Overweight (%)	12.6	69.7	25.0	7.5	10.9	5.0	9.1	17.1	10.5
Obese	2.8 %	18.8 %	0	1.4 %	6.2 %	0	6.2 %	8.0 %	3.1 %
Binge eating									
Overeating (%)	9.8	47.6	0	11.9	14.5	18.6	17.4	13.8	20.0
Binge monthly	1.2 %	7.9 %	0	3.4 %	17.6 %	8.7 %	3.4 %	42.2 %	10.5 %
Binge weekly (%)	0.4	12.2	40.9	1.8	4.7	15.3	1.8	28.0	18.9
Fasting									
Monthly	0.6 %	17.2 %	30.4 %	0.7 %	38.9 %	11.1 %	0.5 %	25.1 %	0
Weekly	0.6 %	29.3 %	69.6 %	0	0.5 %	64.0 %	0	0	100 %
Purging									
Monthly	0.1 %	0	19.0 %	0	61.6 %	6.2 %	2.9 %	6.9 %	5.3 %
Weekly	0	7.8 %	47.6 %	0	6.7 %	59.5 %	0	16.3 %	32.6 %

#### Girls

Amongst girls at age 14 years, Class 1 (94.1 %) identified an asymptomatic group, with low prevalence of engaging in bulimic symptoms and being overweight/obese. Class 2 (prevalence 5.1 %) was characterised by a high percentage of being overweight, overeating or binge eating and engaging in weekly weight control behaviours (WCB). Class 3 (prevalence 0.6 %) resembled bulimia nervosa (BN) and was characterised by a relatively low probability of overweight/obesity and high probability of engaging in weekly WCB and weekly binge eating. At 16 years: Class 1 (non-disordered, 87.0 %): with a low probability of overweight/obesity, of overeating and binge eating, and WCB; Class 2 (prevalence 5.8 %), characterised by a high probability of engaging in monthly WCB and medium probability of engaging in binge eating; Class 3 (prevalence 7.2 %) resembling purging disorder (PD) and/or BN, characterised by a low probability of being overweight/obese and high probability of engaging in weekly WCB. At age 18: Class 1 (non-disordered, 87.8 %) with a low probability of overweight/obesity, of engaging in binge eating, and in WCB; Class 2 (monthly binge eating and WCB) with high prevalence of engaging in monthly binge eating and WCB; Class 3 (weekly WCB, 3.1 %): characterised by low percentages of being overweight/obese (13.6 %) and high probability of weekly WCB.

#### Boys

Due to low prevalence of DE among males, we had small numbers in symptomatic classes amongst boys. At age 14, more than 99 % of the sample was classified as being in the asymptomatic class (class 1) (99.6 %). There were fewer than 10 boys in either of the two symptomatic classes that we identified [Monthly WCB and binge eating (*n* = 7, 0.2 %); Weekly WCB and binge eating (*n* = 6, 0.2 %)], which is smaller than the minimum necessary to draw meaningful inferences. At age 16, Class 1 (non-disordered, 96.5 %) was characterised by a low prevalence of engaging in DE behaviours and low prevalence of overweight/obesity. Class 2, with 79 boys (3.11 %), was characterised by a high prevalence of obesity (77.2 %). Class 3 was small, with only 9 boys (0.4 %) and it was characterised by a large proportion of subjects engaging in weekly WCB (weekly fasting: 77.8 %; weekly purging: 33.3 %). At age 18, Class 1 (non-disordered, 99.6 %) was characterised by low prevalence of overweight/obese (10.8 %) and DE behaviours (binge eating monthly and weekly: 1.8 %; no fasting; purging: 0.3 %); Class 2 (*n* = 6, 0.3 %) albeit smaller in size was comparable to the monthly WCB class at age 14 (monthly binge eating: 33.3 %; monthly fasting: 66.7 %; monthly purging: 83.3 %); Class 3 (*n* = 2, 0.1 %) characterised by high prevalence of weekly weight control behaviours (weekly fasting: 50 %; weekly purging: 100 %).

#### Longitudinal transition

Amongst girls with data on at least two time-points, about 11 % who belonged to the non-disordered class (i.e., had low probability of reporting ED symptoms) were symptomatic at age 16 and/or 18 years (i.e. belonged to symptomatic classes). Between 27.3 and 56.3 % of girls in the two symptomatic classes at age 14 remained in a symptomatic class at age 16; by age 18 a slightly smaller, but comparable, percentage of girls who were in a symptomatic class at 14 years remained in a symptomatic class (21.8 %–41.7 %) (see Table 2). The percentage of girls who were in a symptomatic class (i.e., had a high probability of endorsing ED symptoms) at age 16 years who transitioned to the asymptomatic class by age 18 (was slightly higher compared to the previous time-point (Table 2); between 25.8 and 34.2 % of girls remained in one of the symptomatic classes by age 18 years.

**Table 2:** Proportions of adolescent girls transitioning DE class membership from 14 years to 16 and 18 years (row percentages)

Age 14	Age 16 (*n* = 3115)		
	Non-disordered	Monthly WCB	Weekly WCB
Non-disordered	2601 (88.2 %)	159 (5.4 %)	189 (6.4 %)
Overweight and overeating	109 (72.7 %)	18 (0.3 %)	23 (15.3 %)
Weekly WCB and binge eating	7 (43.7 %)	(12.6 %)^a^	7 (43.7 %)

Age 14	Age 18 (*n* = 2812)		
	Non-disordered	Monthly WCB	Weekly WCB
Non-disordered	2371 (88.9 %)	223 (8.4 %)	73 (2.7 %)
Overweight and overeating	104 (78.2 %)	20 (15.0 %)	9 (6.8 %)
Weekly WCB and binge eating	7 (58.3 %)	(25.0 %)^a^	(16.7 %)^a^

Age 16	Age 18 (*n* = 2718)		
	Non-disordered	Monthly WCB and binge eating	Weekly WCB
Non-disordered	2111 (88.8 %)	223 (9.4 %)	42(1.8 %)
Monthly WCB	115 (74.2 %)	29 (18.7 %)	11 (7.1 %)
Weekly WCB	123 (65.8 %)	36 (19.2 %)	28 (15.0 %)

^a^ *n* < 5

Given the sparsity of boys in symptomatic classes we were unable to investigate transitions amongst boys.

### Adverse outcomes

The odds of adverse psychopathology and behavioural outcomes varied by symptomatic class type in early adolescence (Table 3). Girls in the ‘overweight and overeating’ class had higher odds of anxiety disorders and of any drug use 1.5/2 years later [respectively. OR = 3.26 (95 % CI 1.10–7.87), OR = 2.00 (1.03–3.60)] compared to girls in the non-disordered class. Belonging to the most symptomatic DE class at age 14 was prospectively associated with all adverse outcomes except DSH at 15.5/16 years of age. Due to the low prevalence of this class, our estimates were imprecise as highlighted by the wide 95 % CI (Table 3).

**Table 3:** Multivariable analyses predicting adverse outcomes in ALSPAC girls, odds ratios (95 % CI)

Early adolescence (aged 14 years)	Outcome at later wave of assessment^a^				
	Anxiety disorder (3.3 %)	Depression (28.3 %)	Drug use (7.2 %)	Binge drinking (12.7 %)	DSH (0.7 %)
Available sample size	*N* = 1788	*N* = 2188	*N* = 2162	*N* = 1974	*N* = 2128
Non-disordered	Ref.	Ref.	Ref.	Ref.	–
Overweight and overeating	**3.26 (1.10–7.87)**	1.37 (0.89–2.11)	**2.00 (1.03–3.60)**	1.14 (0.57–2.07)	0.87 (0.56–3.39)
	*p* = 0.02	*p* = 0.14	*p* = 0.028	*p* = 0.69	*p* = 0.89
Weekly WCB & binge eating	**18.46 (2.50–93.65)**	**8.59 (1.52–161.80)**	**17.6 (4.60–71.9)**	**5.86 (1.35–25.47)**	1.83 (0.04–4.24)
	*p* = 0.0009	*p* = 0.046	*p* = 0.00002	*p* = 0.01	*p* = 0.99

Mid-adolescence (aged 16 years)	Outcome at later wave of assessment^b^				
	Anxiety disorder (6.2 %)	Depression (34.4 %)	Drug use (8.4 %)	Binge drinking (54.9 %)	DSH (16.1 %)
Available sample size	*N* = 1,772	*N* = 1,829	*N* = 1,397	*N* = 1,583	*N* = 1,542
Non-disordered	Ref.	Ref.	Ref.	Ref.	Ref.
Monthly WCB	**2.43 (1.43–3.97)**	1.52 (0.99–2.33)	**2.52 (1.20–4.93)**	**2.17 (1.38–3.50)**	**3.34 (2.78–5.92)**
	*p* = 0.0006	*p* = 0.056	*p* = 0.01	*p* = 0.001	*p* = 8.6 × 10^−13^
Weekly WCB	**2.94 (1.84–4.58)**	**1.64 (1.11–2.43)**	1.79 (0.87–3.46)	**1.52 (1.02–2.32)**	**4.06 (2.27–4.91)**
	*p* = 3.7 × 10^−6^	*p* = 0.013	*p* = 0.09	*p* = 0.04	*p* = 3.8 × 10^−10^

Adjusted for age at assessment and occurrence of each outcome at the previous wave, bold indicates *p* < 0.05

^a^Outcomes assessed between age 15.5 and 16 years

^b^Outcomes assessed between age 17.5 and 18 years. The prevalence of each outcome at each timepoint is given in brackets

Girls who belonged to the monthly WCB class at 16 years of age had higher odds of having an anxiety disorder, reporting any drug use, engaging in binge drinking and reporting any episode of DSH at age 17.5/18 compared to girls in the non-disordered class (Table 3).

Similarly to the earlier time-point, girls who engaged in frequent (weekly) WCB at age 16 had higher odds of having an anxiety disorder, depression, engaging in frequent binge drinking and DSH at the following wave (see Table 3).

Amongst boys, we were unable to run predictive models due to small numbers of boys in symptomatic classes. We were unable to investigate adverse outcomes of age 14 DE classes in boys, due to empty cells. Boys in the ‘frequent WCB’ class in mid-adolescence (16 years) were more likely to have depression (77.8 % vs 13.7 %, Fisher’s exact = 10.40, *p* = 0.01), engage in any drug use (22.2 % vs 4.98 %, Fisher’s exact = 6.59, *p* = 0.03) and report any DSH (22.2 vs 4.7 %, Fisher’s exact = 8.9, *p* = 0.02) at 18 years of age.

## Discussion

Amongst adolescent boys and girls from a well-characterised community-based cohort (ALSPAC), we identified a large asymptomatic class and two symptomatic DE classes, which differed in prevalence and characteristics across ages. Amongst girls, symptomatic classes were uncommon at 14 years. Fewer than 5 % of 14 year old girls were classified as being in the class characterised by being overweight and overeating; less than 1 % were in the class characterised by weekly binge eating and weekly WCB (BN-like). At 16 years the size of both symptomatic classes increased. One of the symptomatic classes was characterised by monthly binge eating and WCB, thus resembling OSFED (sub-threshold BN or BED). The other symptomatic class was more mixed, but predominantly characterised by purging behaviour, thus broadly resembling OSFED PD. By age 18 years the latter (BN/PD-like) class was less common, however a symptomatic mixed class of girls engaging in monthly WCB and binge eating (> monthly) became more prevalent (9.1 %).

In relation to transitions, the majority of girls who were highly symptomatic (BN-like class) at age 14 either remained highly symptomatic (BN/PD-like class) or transitioned to the OSFED (sub-threshold BN or BED) class by 16 years. Between age 16 and 18, about 20 % of girls remained in the same class. Similarly to the earlier time-point, transition to the asymptomatic class by age 18 was less common (65.8 vs 72.4 %) for girls in the highly symptomatic class at 16 years.

Belonging to the highly symptomatic (BN-like) class at 14 years was strongly predictive of adverse outcomes (anxiety disorders, depression, drug use and binge drinking) 2 years later, despite wide confidence intervals likely due to small numbers, which resulted in low power to detect differences. Belonging to the BN/PD-like class at 16 years was also associated with later adverse outcomes (anxiety disorders, depression, binge drinking and deliberate self-harm). Both symptomatic classes at age 16 were prospectively associated with adverse outcomes 2 years later, suggesting that higher (weekly) vs. lower (monthly) frequency of ED behaviours might not necessarily index worse outcomes.

Despite our large sample, analyses on boys were underpowered, due to low numbers of symptomatic individuals. Therefor,e we performed exploratory analyses that should not be overinterpreted. Amongst boys, symptomatic classes had very low prevalence, and across all three ages a stable class characterised by frequent WCB was evident. Belonging to this class at 16 years was associated with later depression, drug use and DSH.

Similar to our previous study [18] and others [16] this study highlights that a sizeable percentage of girls in the community across early, mid and late adolescence can be grouped into categories defined by frequent ED symptoms. Classes identified in this study were not fully consistent with DSM-5 categories; although an uncommon BN-like class was evident at 14 years, at later time-points this highly symptomatic class seemed to represent youth with frequent compensatory behaviours (independent of frequent binge eating). This might suggest that individuals with BN and purging type disorders in the community are similar in characteristics; else, due to our sample size rare disorder-specific classes might have been ‘forced’ into one larger class.

Consistent with findings from Kansi et al. [16] we found that classes were not stable over the 4 years under study, in particular both studies highlight a transitory increase in prevalence of bulimic symptomatology classes around mid-adolescence (16 years). In our sample ~50 % of these girls transitioned to an asymptomatic class by age 18, suggesting remission (whether natural or treatment-driven is not possible to detail in our sample), or a temporary decrease in symptomatology.

Consistent with our previous findings [18] and other studies [36, 37] high levels of symptoms both in early- and mid-adolescence were prospectively associated with higher odds of psychopathology and behavioural outcomes. Due to the low prevalence of the BN-like class at age 14 and low precision of our estimate we cannot discern whether the odds of adverse outcomes differed across ages. However, similar to our previous studies [2, 4, 18] frequency of DE behaviours did not seemingly differentiate prognosis in terms of the outcomes under study.

These findings should be considered in the context of strengths and limitations. We relied on a relatively large sample of youth that have been well characterised over the years. We were able to use data collected at three specific time-points in adolescence covering a span of about 4 years. Due to the nature of ALSPAC, i.e., a birth cohort, the narrow age range at each assessment ensures good homogeneity at each time-point. Another strength is that many of the outcomes under study, as well as some of the indicators (e.g., BMI), were obtained from interviews or face-to-face assessments rather than questionnaires. Important limitations to highlight, however, relate to attrition, which was relatively high at age 18, and was higher amongst girls who were assigned to symptomatic classes vs. those in the asymptomatic class at 14 years of age; low power to investigate boys, due to low endorsement of ED symptoms under study. This might be due to the questions included as indicators, maybe geared towards female-specific ED behaviours and cognitions [38, 39]. Indicators used were based on questions derived from the Youth Risk Behaviour Surveillance System questionnaire [22], therefore symptoms were self-reported and fewer behaviours consistent with anorexia nervosa were present in the questionnaire, therefore limiting our ability to investigate anorexia-type behaviours. Data on pubertal status or menstrual abnormalities (for girls) concurrent to outcome assessment were not available to the authors. Although ALSPAC is representative of the area of south-west England the sample is drawn from, generalizability to the whole of the UK might be limited by relatively low representation of ethnic minorities.

## Conclusions

Our findings indicate that in a community sample of UK adolescents groups of girls that engage in DE symptoms can be identified across ages. These girls are not only likely to have persistent symptoms, but they are also more likely to present higher levels of psychopathology and worse behavioural outcomes at later ages compared to their peers. Although diagnostic classification has been revised to relax thresholds, our study suggests that a group of girls who engage in ED behaviours at lower frequency than specified by DSM-5 criteria (and likely ICD-11) have negative outcomes. These girls may be unlikely to seek treatment, or be recognised as having DE, and therefore might not be identified as benefitting from early intervention in a clinical setting. Public health interventions would therefore be very relevant for this group.

There was some evidence of DE classes instability in mid-adolescence, with a decrease in prevalence of symptomatic classes by age 18, suggesting ED symptoms might remit or improve in late adolescence. These findings need further investigation to understand potential protective factors for persistence of symptoms. Lastly although we were only able to explore empirically derived classification in boys, this study offers some preliminary findings on a small group of boys who engage in WCB and might be at risk for later adverse outcomes.
